# The effects of communicating uncertainty around statistics, on public trust

**DOI:** 10.1098/rsos.230604

**Published:** 2023-11-22

**Authors:** John Kerr, Anne-Marthe van der Bles, Sarah Dryhurst, Claudia R. Schneider, Vivien Chopurian, Alexandra L. J. Freeman, Sander van der Linden

**Affiliations:** ^1^ Winton Centre for Risk & Evidence Communication, University of Cambridge, Cambridge CB2 1TN, UK; ^2^ Department of Psychology, University of Cambridge, Cambridge CB2 1TN, UK; ^3^ Department of Public Health, University of Otago, Wellington, New Zealand; ^4^ Department of Social Psychology, The University of Groningen, Groningen, Netherlands; ^5^ Department of Psychology, Humboldt-Universität zu Berlin, Berlin 10099, Germany

**Keywords:** uncertainty, communication, COVID-19, trust, trustworthiness

## Abstract

Uncertainty around statistics is inevitable. However, communicators of uncertain statistics, particularly in high-stakes and potentially political circumstances, may be concerned that presenting uncertainties could undermine the perceived trustworthiness of the information or its source. In a large survey experiment (Study 1; *N* = 10 519), we report that communicating uncertainty around present COVID-19 statistics in the form of a numeric range (versus no uncertainty) may lead to slightly lower perceived trustworthiness of the number presented but has no impact on perceived trustworthiness of the source of the information. We also show that this minimal impact of numeric uncertainty on trustworthiness is also present when communicating future, projected COVID-19 statistics (Study 2; *N* = 2,309). Conversely, we find statements about the mere existence of uncertainty, without quantification, can reduce both perceived trustworthiness of the numbers and of their source. Our findings add to others suggesting that communicators can be transparent about statistical uncertainty without undermining their credibility as a source but should endeavour to provide a quantification, such as a numeric range, where possible.

## 1. Introduction

For those making evidence-informed decisions, the trustworthiness of each piece of evidence influences how much weight is put on that information in the decision-making process. Research shows that the level of trust that people say they have in the source of the information, or how trustworthy they report the information itself to be, are associated with people's decisions and support for policies in relation to that information [1–4].

One important aspect that may affect people's perception of the trustworthiness of information, or its source, is the degree of uncertainty they perceive around it and how much of that has been communicated. Uncertainty is unavoidable: the precision and reliability of scientific evidence, for instance, is always limited by our inability to observe all relevant factors, our methodology or systematic biases [5,6]. Further uncertainty may also arise, especially when we are considering projections into the future, from randomness and chance [7–9]. The magnitude of the uncertainty around a fact or number *should* affect an audience's perception of its accuracy and may therefore change the weight they put on it in a decision-making process [10,11]. However, some researchers and communicators express broader concerns, that communication of uncertainties could undermine audiences' perception of the trustworthiness of the communicator and information quality [6,12–18]. Concerns are also sometimes expressed about uncertainties being manipulated by sceptics or political opponents to decrease trust in scientists, challenge policy makers or cast doubt on the information itself [19–22]. On the other hand, given that the trustworthiness of a communicator is associated with honesty, openness and integrity [23,24] being transparent about uncertainty might contribute to building trust rather than undermining it [25,26], especially in the face of changing information [27].

The difficulties of making high-stakes decisions under uncertainty became especially apparent to both policymakers and the public during the COVID-19 pandemic, when important decisions were having to be made daily despite considerable uncertainty about the facts and statistics, and putting off a decision until more information was available was not possible (or itself had considerable consequences) [28]. The outbreak of the COVID-19 pandemic, then, provided an excellent case study to investigate the effects of the communication of uncertainties on the perceived trustworthiness of information and the source of the information. Our focus on perceptions of trustworthiness in the current research, as opposed to trust, arises from a key distinction between the two. Trustworthiness can be considered a property of the communicator, while putting trust in someone or something is an act of the receiver [29]. We aim to investigate effects on the communicator side, both in terms of how information that is communicated as well as its source is perceived. While we use this framing, we note that there is variation in the literature and many of the studies cited below examine related concepts of trust, credibility and reliability.

There are many kinds of uncertainty, and they may have different effects on the audience (see [9] for a review). Van der Bles *et al*. [9] distinguish between ‘indirect’ and ‘direct’ uncertainty. Indirect uncertainty is uncertainty about the evidence behind a number or claim, and according to the authors, ‘will generally be communicated as a list of caveats about the underlying sources of evidence, possibly amalgamated into a qualitative or ordered categorical scale.’ (p. 7) Direct uncertainty—uncertainty about the number or claim itself (given the evidence)—is typically expressed in numeric form, such as an uncertainty interval or probability distribution, or ‘given an approximate quantitative form, verbal summary and so on’ (p. 7). This is distinct from the often-used categories of ‘epistemic’ and ‘aleatory’ uncertainty. These terms distinguish uncertainty which could in theory be reduced through more information (epistemic uncertainty) from ‘irreducible’ uncertainty arising from stochastic processes (aleatory uncertainty). In this study we concentrate on ‘direct’ uncertainty, but even different ways of representing this can have different effects.

Specifying the numerical range around a point estimate, such as confidence intervals, signals to the lay reader that the estimate is not certain but places bounds on how high or low the actual figure may reasonably be. However, numerical ranges can be hard to put into context for non-experts and their interpretation can be biased by prior beliefs [9,30,31]. Direct uncertainty can also be expressed in a verbal format. For example, a communicator might say a statistic is ‘about’ a certain number, or, being more explicit, state ‘there is some uncertainty around the estimate, it could be somewhat higher or lower’. Such language can express the fact that an estimate is not certain without the use of numbers and statistical ranges and might therefore be more understandable to non-experts. However, these expressions might come with their own issues, such as ambiguity and a lack of precision.

Previous research suggests mixed results for the effect of communicating uncertainty in numerical formats on perceptions of trustworthiness. Some studies comparing statistics presented with or without numeric uncertainty find no significant effect on trust in the information and scientific beliefs [32,33]. Others suggest a positive effect on ‘perceived honesty’ for most participants, but a negative effect for a small subset of participants [34]. One study on COVID-19 statistics [28] reports that participants presented with an estimate of future COVID-19 deaths expressed less general trust in scientists when that estimate was provided as a range rather than a single number. The authors conclude that expressing uncertainty as a range: ‘may be more intellectually honest, but it nonetheless comes at a cost of eroding public confidence’ (p. 5).

Acknowledging uncertainty in a verbal, rather than numeric, format has also been studied in different ways, and results vary depending on the method. For example, inclusion of words such as ‘estimated’ or ‘around’, as an expression of verbal uncertainty, had no effect on trust in a number or of the source of that number, when the number was presented in the context of a statement or news article [6]. A similar study found that such hedging terms, for instance ‘may’ and ‘possibly’, increased participants' perception of the complexity of statements, but had no bearing on the perceived credibility, ‘scientific-ness’ or strength of the arguments presented [35].

Statements which are more explicit in acknowledging (unquantified) uncertainty with words (e.g. ‘We are not exactly sure how effective [an influenza vaccine] will be’) can lead to lower trust in communicators compared to statements with no uncertainty [14]. Similarly, phrases such as ‘there is uncertainty around the exact figure—it could be higher or lower' decrease peoples’ perceptions of the trustworthiness of the information and source, relative to statements without uncertainty [6]. However, an experiment in which participants read statements about current estimates of UK GDP reports differing results. Including the caveat ‘this estimate is likely to be revised as updated information becomes available’ (versus not) was reported to have no effect on trust in the source of the information [36].

In the context of the current studies, we focus on these more explicit statements about the mere existence of uncertainty with no accompanying numeric information, which we label as ‘verbal uncertainty’ [6]. We acknowledge that elsewhere in the literature the term verbal uncertainty has been applied to hedge words (e.g. ‘approximately’), or verbal probabilities (e.g. ‘likely’). We also note that our verbal uncertainty manipulation in the experiments below also contains ambiguity (or vagueness) with no indication of magnitude, and results should be interpreted with this in mind (cf. [37]).

Apart from communication format, the timeframe of the statistics communicated may be consequential for perceptions of trustworthiness: do claims relate to the past, present or future? This point is of importance as it is possible that uncertainty about future events may be treated differently to uncertainty about past or present events, which in theory could be known. Claims about the future are generally recognized to be more uncertain than claims about the present [38]. For example, people perceive claims about the state of the climate in 100 years' time to be more uncertain than claims about the climate in the next year [39]. On the one hand, greater perceived uncertainty about future versus current events (independent of any communicated uncertainty) may lead to lower perceptions of trustworthiness or exacerbate negative effects of communicated uncertainty on trustworthiness. This view is supported by research showing that the negative effects of uncertainty information on trust in information are in part mediated by perceptions of uncertainty [6,40]. As put by the authors: ‘this suggests that the more uncertain people perceive the numbers to be, the less reliable and trustworthy they find them.’ ([6] p. 7680).

On the other hand, people may view uncertainty about future outcomes as more acceptable or, to use the term broadly, forgivable. For example, they may be more likely to ascribe communicated uncertainty about future outcomes to the inherent nature of the future rather than the incompetence or ignorance of the source of the information (as suggested by [41]). And therefore, less likely to downgrade the level of trust placed in the source of the information.

Notably, in experiments investigating the effects of both numeric and verbal uncertainty reviewed above, there is variation in terms of whether the central information communicated to participants relates to current knowledge (e.g. [5]) or future predictions (e.g. [14,28]). To our knowledge, no experimental research has examined the potential moderating role of timeframe (i.e. claims about current knowledge versus future predictions) on the effects of communicating numeric and verbal uncertainty. As articulated by Dieckman *et al*. [41], ‘Members of the public may have fundamentally different perceptions of whether or how experts answer what-will-happen questions as compared to what-is questions.’ (p. 334) If the temporal framing (present versus future) of communicated statistics influences the effects of uncertainty information on perceptions of trustworthiness, this would have practical and theoretical implications. For communicators of statistics, this would introduce an additional factor for consideration if deliberating on how to communicate uncertainty while maintaining trustworthiness in the eyes of an audience. From a theoretical perspective, the distinction between present versus future claims may offer insights into the mixed findings of the literature. Reviews of the effects of uncertainty communication on trustworthiness and credibility have not always distinguished between claims regarding the present and the future predictions (e.g. [5]).

Of interest in the current studies is the extent to which numeric and verbal expressions of uncertainty are *perceived as uncertain* relative to statements without uncertainty. Following from [6], we also consider how uncertain participants perceived the information to be, in addition to measuring perceived trustworthiness of the information and of the source.

In this set of studies, we took advantage of the opportunity to insert an experiment within international surveys fielded in the first months of the COVID-19 pandemic, to assess the effects of verbal and numerical formats for the communication of statistical uncertainty. Our aim was to see how previous findings in the literature would play out in the context of COVID-19 statistics (at this early stage of the pandemic), in an international sample. Then to extend this research to investigate any potential differences between the perception of communication of uncertainty in the context of present knowledge and that of future projections.

We carried out the first experimental study using populations from 12 countries across Europe, North and Central America, and Asia, quota-sampled to be representative on age and sex (Study 1). Participants were randomized to one of three uncertainty communications conditions (format: control, numerical or verbal uncertainty) about current COVID-19 hospitalization rates (i.e. exploring uncertainty around present knowledge only). In Study 2, carried out in a UK population, representative on age and sex, we further explore the effect of uncertainty format while varying the temporal frame of statistics. In a crossed experimental design, participants were presented with estimates of either *current* or *predicted future* COVID-19 deaths, which included either no uncertainty (control), numeric uncertainty or verbal uncertainty.

Both studies address the research question: does communication of numeric or verbal uncertainty (versus no uncertainty) around COVID-19 statistics influence the perceived trustworthiness of the information or its source? Study 2 also addresses the following research question: do these effects differ if statistics relate to future projections rather than current estimates?

## 2. Study 1

### 2.1. Methods

Participants were recruited across 12 countries. Participants in Australia were recruited through Dynata (dynata.com), participants in France were recruited by BVA (bva-group.com) and US and UK samples were recruited via Prolific (prolific.co). Participants in all other countries were recruited through Respondi (respondi.com). Participants were paid for their participation (between £1-3GBP, depending on recruitment platform and location). Data collection was carried out between March and May 2020, with each survey fielded for approximately five days. Interlocking quotas were used to match samples to the national profile on age and sex (and ethnicity for Prolific samples). Sample sizes and characteristics are reported in table 1. The French sample was collected in a survey carried out for other research and the sample size reflects the needs of that research. Precise information on response rates was not provided by panel providers, however Respondi reports that response rates for US and UK invitees are typically around 30%. The translation process for non-English surveys was limited by the resourcing and time available at that stage of the pandemic. Surveys were translated from English to other languages by native speakers fluent in English and familiar with the project and aims. Difficult terms were discussed with additional native speakers and the team to determine the best choice of words.

**Table 1.** Samples included in Study 1.

sample	*N*	female (%)	mean age	s.d.
Australia	672	52.08	46.31	16.44
China	699	48.78	43.24	14.26
France	3002	52.47	48.79	16.53
Germany	688	49.13	46.71	15.92
Italy	618	51.62	45.91	14.81
Japan	700	51.00	48.00	16.23
South Korea	700	49.00	45.26	15.38
Mexico	661	50.53	38.99	14.18
Spain	690	50.87	46.68	14.99
Sweden	684	48.54	45.49	16.02
UK	703	50.92	45.63	15.69
US	702	50.57	45.14	15.84
Total	10519	50.89	46.26	15.97

The study was approved by the University of Cambridge's Psychology Research Ethics Committee (PRE.2020.034).

A Gpower [42] power calculation indicated that sample sizes were sufficient to detect effect sizes reported in previous research, e.g. a *d* = 0.55 effect of verbal uncertainty (versus control) on perceived trustworthiness of the numbers at 95% power and an alpha level of 0.05 (based on the internal meta-analysis in [6]).

Participants completed an online survey experiment embedded in a larger survey including questions on COVID-19 risk perceptions and attitudes, hosted on the Qualtrics survey platform. The surveys themselves can be viewed in the OSF project https://osf.io/jnu74/ (the English language version is dated 19^th^ March 2020 and labelled ‘US’). In the survey, participants were randomly assigned to one of three ‘format’ conditions. All participants were shown a piece of text about COVID-19: ‘*Illness due to COVID-19 infection is generally mild, especially for children and young adults. However, it can cause serious illness: for people aged 70–80, about 17% of those who catch it need hospital care.’* In the control format condition this was the exact text shown. In the ‘numeric uncertainty’ format condition, the text shown to participants additionally had the phrase ‘*(range between 10% and 34%)*’ inserted after the percentage figure. In the ‘verbal uncertainty’ format condition, the text shown to participants included the additional sentence ‘*There is some uncertainty about that percentage, it could be somewhat higher or lower*’ at the end (but no numerical range was shown). The word ‘about’ in the control condition was included in order to make the sentence read naturally to participants in all conditions, following a common pattern in expressing probabilities, and in line with similar previous studies [6,36]. This phrasing was consistent across all experimental messages. Previous experiments using such single-word hedges in the context of a whole statement suggest that they do not affect participants' perceptions of the uncertainty or credibility of the fact or statement [6]. The infection hospitalization rate and associated uncertainty interval presented in stimuli were sourced from Verity *et al*. [43], which provided the first robust, peer-reviewed estimates of age-stratified infection hospitalization rates and was published just prior to data collection.

Participants were then asked a series of questions forming our main dependent variables. Perceived uncertainty was measured with the item ‘*To what extent do you think this number is certain or uncertain?*’ (responses: 1 = ‘*Very certain*’ to 7 = ‘*Very uncertain*’). Perceived trustworthiness of the number was measured as the average of three items: ‘*To what extent do you think this number is reliable?’, ‘To what extent do you think this number is accurate?’,* and *‘To what extent do you think this number is trustworthy?’* (1 = ‘*Not at all*’ to 7 = ‘*Very*’, Cronbach's *α* = 0.93, individual country *α*s: 0.90–0.96).^1^ Perceived trustworthiness of the source of the information was measured with the item ‘*To what extent do you think that the people responsible for producing this number are trustworthy?’ (1 = ‘Not at all’* to 7 = ‘*Very’*). Participants also answered questions about how positive or negative they felt about the information and how easy they found it to read. Details and results for these secondary outcomes and correlations between dependent variables (at the individual and country-mean level) are reported in the supplementary material (electronic supplementary material, Appendices S1 and S2).

#### 2.1.1. Analytic approach

To examine the effect of uncertainty information on perceptions of uncertainty and number and source trustworthiness, accounting for variation between countries, we used a linear mixed modelling approach. This allows estimation of the overall effects of the experimental manipulation as well as offering insight into inter-country variation in terms of overall rating of messages (via random intercepts) and experimental effects (via random slopes).

For the model predicting perceived uncertainty, modelling random slopes in addition to random intercepts resulted in a singular fit and estimation of random effect correlations close to −1, indicating overfitting and a failure to converge. To circumvent this, we refitted the model with random effect correlations suppressed to reduce model complexity [44,45]. The same issue was also encountered with the model predicting number trustworthiness, and the same modification applied. In the case of the model predicting source trustworthiness, singular fit also occurred and was not resolved by supressing random effect correlations. This was most likely due to a lack of variance in random slopes. Following the advice of [45], we report here the random intercepts only model, which did not result in a singular fit. Notably, none of these decisions had a substantial impact on the magnitude of the fixed effects (see analysis files on OSF repository: https://osf.io/y982k/).

### 2.2. Results

**Figure 1.**
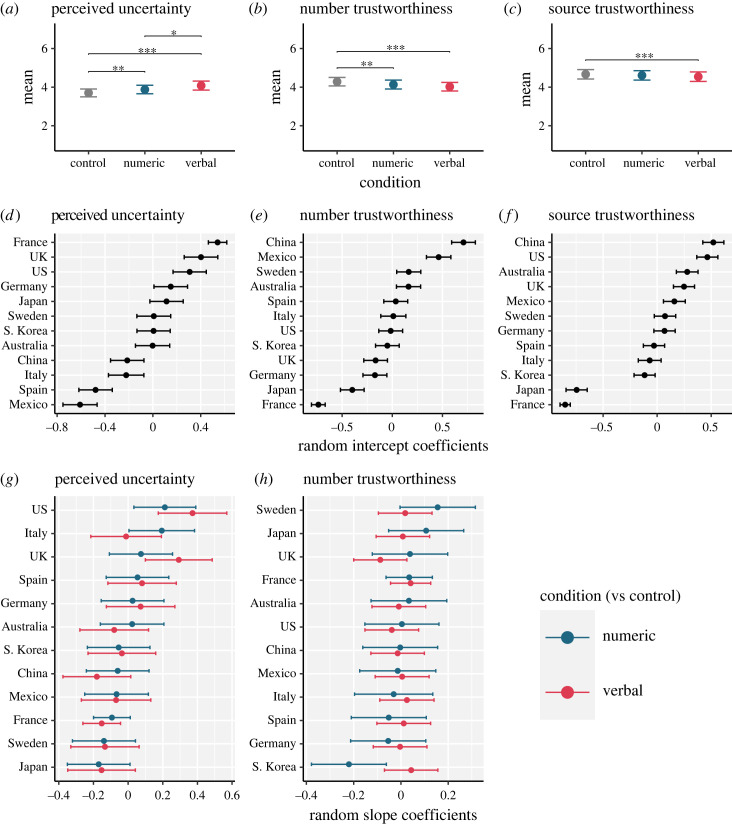
Visualization of mixed model results. Panels (*a–c*) present marginal means predicted by models reported in table 2. Means and 95% CI shown with brackets and asterisks indicating significant pairwise differences based on Tukey *post hoc* tests (***p < 0.001, **p < 0.01, *p < 0.05). Panels (*d–f*) show by-country random intercept coefficients with 95% CI. Panels (*g,h*) show by country random slope coefficients for the effect of numeric (blue) and verbal uncertainty (red) messages with 95%CI.

We report the model results in table 2 (*p*-values computed using a Wald t-distribution approximation and *R*^2^ values based on [46] as implemented by the *sjPlot* R package [47]). Fixed effects are visualized in figure 1*a–c* as estimated marginal means with *post-hoc* pairwise comparisons (computed using the *emmeans* R package [48]). We also display the by-country random intercept coefficients from these models in figure 1*d–f*. These provide an indication of which countries tended to rate messages higher or lower on a given outcome, relative to the fixed intercept. Lastly, for perceived uncertainty (figure 1*g*) and number trustworthiness (figure 1*h*) we plot the by-country random slope coefficients for the effect of the numeric uncertainty message (versus control; blue points) and the verbal uncertainty message (versus control; red points). These provide an indication of in which countries experimental effects were larger or smaller, relative to the fixed effects. For completeness we plot all means and pairwise comparisons, at the country level, in the supplementary material (electronic supplementary material, appendix S3).

**Table 2.** Results of mixed models investigating the effect of message condition on outcomes.

predictors	1. perceived uncertainty			2. number trustworthiness			3. source trustworthiness		
	*b*	95CI	*P*	*b*	95CI	*p*	*b*	95CI	*p*
(intercept)	3.70	3.50–3.91	<0.001	4.28	4.06–4.50	<0.001	4.67	4.42–4.91	<0.001
numeric	0.17	0.07–0.28	0.002	−0.15	−0.24 – −0.05	0.002	−0.06	−0.12–0.01	0.075
verbal	0.38	0.24–0.51	<0.001	−0.26	−0.34 – −0.19	<0.001	−0.12	−0.19 – −0.06	<0.001
random effects									
*σ*^2^	1.73			1.69			1.76		
*τ*_00_	0.12 _Country_			0.14 _Country_			0.18 _Country_		
*τ*_11_	0.02 _Country.Numeric_			0.01 _Country.Numeric_					
	0.04 _Country.Verbal_			0.00 _Country.Verbal_					
ICC	0.07			0.08			0.09		
*N*	12 _Country_			12 _Country_			12 _Country_		
observations	10475			10457			10470		
*R*^2^_Marginal_	0.013			0.006			0.001		
*R*^2^_Conditional_	0.077			0.084			0.094		

Unstandardized effects shown. 95CI = 95% confidence interval, *σ*^2^ = residual variance, *τ*_00_ = random intercept variance, *τ*_11_ = random slope variance, ICC = Intraclass Correlation Coefficient.

We outline key model results for the three outcomes examined in the sections below.

#### 2.2.1. Perceived message uncertainty

Fixed effects for the experimental manipulation contrasts indicated that perceived uncertainty was, on average, higher for participants in the numeric (*b* = 0.17, 95% CI [0.07, 0.28], *β* = 0.13, *p* = 0.002) and verbal conditions (*b* = 0.38 [0.24, 0.51], *β* = 0.28, *p* < 0.001) compared to the control condition (table 2, Model 1). However the intercept (analogous to perceived uncertainty of the control message) and the strength of these effects varied across countries. Random intercept coefficients are shown in figure 1*d*. Broadly speaking, participants in France, the US and UK, on average, rated messages as more uncertain than other countries, while China, Italy, Spain and Mexico tended to rate messages as less uncertain. The effect of numeric uncertainty (versus control) also varied by country, with larger effects in US and Italian samples. Random slope coefficients also indicate that the effect of the verbal uncertainty message (versus control) was larger in the US and UK, and smaller in France.

#### 2.2.2. Number trustworthiness

As shown in table 2, Model 2, fixed effects for the experimental manipulation contrasts indicated that, on average, participants in the numeric and verbal conditions (versus control) rated the message read as less trustworthy (*b*_Numeric_ = −0.15 [−0.24 – −0.05], *β* = −0.11, *p* = 0.002; *b*_Verbal_ = −0.26 [−0.34 – −0.19], *β* = −0.19, *p* < 0.001). However, the intercept and the strength of these effects varied across countries. Random intercept coefficients are shown in figure 1*e*. Results indicate that participants in China, Mexico, Sweden and Australia rated the numbers presented as more trustworthy relative to the fixed effects, while France, Japan, Germany and UK rated numbers as less trustworthy. Random slope estimates in figure 1*h* and estimated variance (table 2) indicate little by-country variation in the effect of either the numeric or verbal condition (versus Control). The one exception was South Korea, where the random slope coefficient indicated that the negative effect of the Numeric condition on trustworthiness was greater, relative to the fixed effects noted above.

#### 2.2.3. Source trustworthiness

Considering perceived trustworthiness of the source of the information, fixed effects from a random intercepts model (table 2, Model 3) indicated that the verbal condition (versus control) had a negative effect (*b* = −0.12 [−0.19 – −0.06], *β* = −0.09, *p* < .001), while the effect of the numeric condition was not significant. There was by-country variation in terms of random intercepts (summarized in figure 1*f*) with Japan and France in particular more likely to rate the message source as less trustworthy in general. As noted above, a random slopes model could not be fitted for this outcome due to encountering singular fit.

### 2.3. Interim discussion

This large dataset broadly confirms the findings of our previous work in other contexts [6]. While presenting uncertainty information around a percentage increased perceived uncertainty around the reported hospitalization rate, it led only to a small decrease in perceived trustworthiness of the numbers. The explicit verbal uncertainty statement decreased trustworthiness in the number more than numeric uncertainty. Considering perceived trustworthiness of the *source* of the numbers, participants were less trusting of the source when verbal uncertainty was communicated (versus control condition). However, this small effect was only detectable in a pooled sample combining all countries. Uncertainty expressed in the form of a range had no significant impact on the trustworthiness of the source.

There was variation between countries in terms of participants' baseline ratings of messages (random intercepts), and to some extent the effect of the numeric and verbal uncertainty on perceptions of uncertainty. Considering the perceived trustworthiness of the numbers presented in the message, we find surprisingly little by-country variation in terms of experimental effects (random slopes). In this study the uncertainty presented was purely a result of incomplete knowledge about present hospitalization rates. To test whether the findings extended to the public's reaction to uncertainty about the future (which brings in elements of randomness and unpredictability) we carried out a further study within the UK only.

## 3. Study 2

The design of this study followed that of Study 1, with the addition of a further experimental factor that varied whether the information participants read related to the present or future.

### 3.1. Methods

A total of 2,309 UK adult participants (51.4% female; *M*_age_ = 45.2, *s.d.* = 15.8) were recruited in between 7^th^ and 10^th^ of May, 2020 via online panel providers Respondi (*n* = 1150) and Prolific (*n* = 1159). Sample size was determined by the requirements of a larger sub-study in the context of a wider ongoing study on COVID-19 attitudes and behaviours. A conservative sensitivity analysis revealed that the smallest pairwise effect detectable at a sample size of *n* = 2,309 across the experimental groups and 95% power at alpha level 0.05 was *d* = 0.26. Quotas were set to match the participant pool to the national profile on age, sex, and, for the Prolific sample, ethnicity. Participants were paid approximately £2GBP for their participation. The study was approved by the University of Cambridge's Psychology Research Ethics Committee (PRE.2020.034).

As in Study 1, participants completed an online survey experiment on the Qualtrics platform. The experiment was embedded in a larger survey which included additional items relating to COVID-19 attitudes and behaviour not reported here. Participants were randomized to one of six conditions, in a 3 (uncertainty format)×2 (uncertainty type) factorial experiment.

The stimuli in this experiment were drawn from statistics reported by modelling from Imperial College London (ICL) [49]. Specifically, estimates of current deaths were taken from data provided on the ICL COVID-19 modelling website [50]. Forecast daily deaths were based on the median estimate and 95% credible interval produced by an ICL ensemble model drawing on four separate models, calculated on the 4^th^ May, 2020 [51]. To ensure that the participants were not misled by using factually inaccurate statistics on such an important topic, real modelling figures had to be used, reflecting the actual state of the pandemic at that time in the UK. This ethical imperative does introduce a confound to the experimental design as there was necessarily a difference in point estimates and intervals presented to participants in the different uncertainty type conditions. We return to this point in discussing the results.

Those in the present uncertainty condition were given the core information ‘*Looking at the average number of deaths per day over a period of time helps to understand whether the COVID-19 epidemic is stabilizing in the United Kingdom. This past week, the average number of deaths in the UK was about 555 per day*.’ Control participants received only this information. Those in the numeric uncertainty format condition were given the extra information ‘*(range between 439 and 698).*’ Whilst those in the verbal uncertainty format condition were instead given the extra information ‘*There is some uncertainty about this number, it could be somewhat higher or lower*.’

Those in the future uncertainty condition were given the information ‘*Looking at the average number of deaths per day over a period of time helps to understand whether the COVID-19 epidemic is stabilizing in the United Kingdom. This upcoming week, the average number of deaths in the UK is expected to be about 591 per day*.’ Control participants received only this information. Those in the numeric uncertainty format condition were given the extra information ‘*(range between 371 and 1081).*’ While those in the verbal uncertainty format condition were instead given the extra information ‘*There is some uncertainty about this number, it could be somewhat higher or lower.*’

Participants were then asked the same questions as in Study 1 to ascertain their perception of the uncertainty in the number, the perceived trustworthiness of the number (three items; *α* = .94) and the perceived trustworthiness of the people responsible for producing the number. Participants also answered questions about how positive or negative the information made them feel, how easy it was to understand and how competent they thought the source of the information was. Details and results for these secondary outcomes, correlations between all outcomes are reported in the supplementary material (electronic supplementary material, Appendices S4 and S5).

### 3.2. Results

Considering first *perceived uncertainty*, a two-way ANOVA examining the effect of uncertainty format (control, numeric or verbal) and uncertainty type (present or future), revealed a significant interaction, *F*_2, 2299_ = 4.01, *p* = 0.02, *η_p_*^2^ = 0.003, indicating that the effect of format differed across uncertainty types. This was followed up with one-way ANOVAs examining the effect of format in each uncertainty type group. Format had a significant effect on perceived uncertainty among people who read a message with present uncertainty, *F*_2, 1156_ = 42.25, *p* < 0.001, *η_p_*^2^ = 0.068, and smaller but still significant effect for those who read a message with future uncertainty, *F*_2, 1143_ = 15.24, *p* < 0.001, *η_p_*^2^ = 0.026. We report post hoc analyses of the effect of format for each of these uncertainty types (present or future) in turn below.

Post hoc tests revealed that, among participants presented with present uncertainty, verbal uncertainty (*M*_verbal_ = 4.54, *SD* = 1.53) was perceived as more uncertain than numeric uncertainty (*M*_numeric_ = 3.70, *SD* = 1.36; *p* < 0.001, *d* = 0.58) or the control (*M*_control_ = 3.72, *SD* = 1.39; *p* < 0.001, *d* = 0.55). There was no significant difference between uncertainty ratings in the control and numeric conditions (*p* = 0.98, *d* = 0.01).

**Figure 2.**
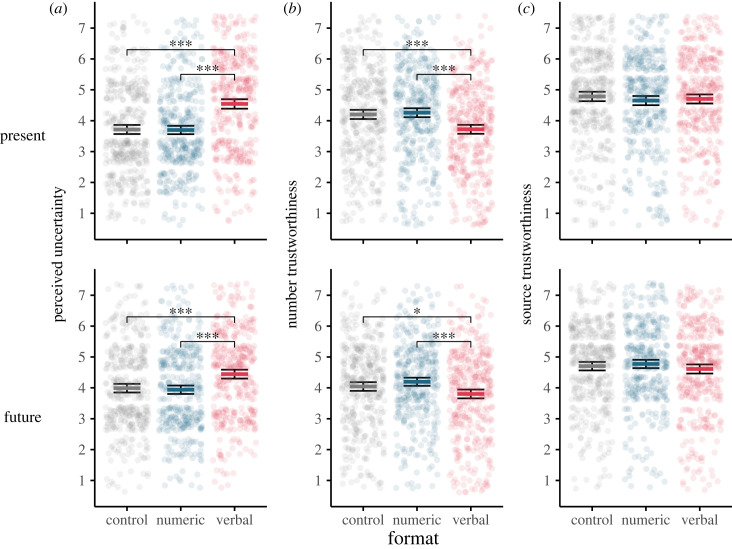
The effect of uncertainty format and type on: (*a*) perceived uncertainty, (*b*) perceived trustworthiness of numbers, and (*c*) perceived trustworthiness of source (means and 95% CI). Means for present and future uncertainty conditions shown in upper and lower panels respectively. Jittered points display the underlying data with each point representing an individual response. Vertical displacement is ±40% the resolution of the data, so for integer scales (perceived uncertainty and source trustworthiness) points are vertically displaced by up to ± 0.4 to display density without over-plotting. Horizontal brackets indicate a significant pairwise difference between format conditions, *p < 0.05, ***p < 0.001.

A similar pattern was seen among those presented with future uncertainty, though the effects were smaller: verbal uncertainty (*M*_verbal_ = 4.44, *SD* = 1.42) was perceived to be significantly more uncertain than numeric uncertainty (*M*_numeric_ = 3.94, *SD* = 1.35; *p* < 0.001, *d* = 0.36) or the control (*M*_control_ = 3.99, *SD* = 1.39; *p* < 0.001, *d* = 0.23). There was no significant difference between uncertainty ratings in the control and numeric conditions (*p* = 0.87, *d* = 0.04). Pairwise differences are displayed graphically in figure 2*a*.

We also examined pairwise differences between present and future uncertainty types within each of the three format groups (means reported above). Among participants in the control format condition (who read no additional uncertainty information), uncertainty was perceived to be higher in the future group than the present group (*p* = 0.008, *d* = 0.19). Among participants presented with the numeric format, uncertainty was also perceived to be higher in the future group than present group (*p* = 0.014, *d* = 0.17). In the verbal uncertainty condition, there was no significant difference in perceived uncertainty between future and present groups (*p* = 0.36, *d* = 0.07).

Considering *perceived trustworthiness of the numbers*, a two-way ANOVA found no significant main effect of uncertainty type (i.e. no significant difference between present and future uncertainty across formats; *F*_1, 2302_ = 0.62, *p* = 0.43, *η_p_*^2^ < 0.001). We did find a significant main effect of uncertainty format, *F*_2, 2302_ = 22.69, *p* < 0.001, *η_p_*^2^ = 0.019. There was no significant interaction effect, *F*_2, 2302_ = 1.40, *p* = 0.25, *η_p_*^2^ = 0.001.

*Post-hoc* tests comparing between format conditions (pooled across present and future uncertainty types) revealed that participants who read an explicit verbal uncertainty cue considered the number less trustworthy compared to participants who read a numeric uncertainty cue (*M*_numeric_ = 4.23, *SD* = 1.38; *M*_verbal_ = 3.76, *SD* = 1.43; *p* < 0.001, *d* = 0.33) or the control condition (*M*_control_ = 4.12, *SD* = 1.45; *p* < 0.001, *d* = 0.25). There was no significant difference between control and numeric conditions (*p* = 0.32, *d* = 0.07) (figure 2*b*).

Lastly, considering *perceived trustworthiness of the source* of the information, a two-way ANOVA showed no significant effects of uncertainty type (*F*_1, 2299_ = 0.08, *p* = 0.77, *η_p_*^2^ = 0.001), of uncertainty format (*F*_2, 2299_ = 0.70, *p* = 0.50, *η_p_*^2^ < 0.001) or their interaction (*F*_2, 2299_ = 1.37, *p* = 0.26, *η_p_*^2^ = 0.001): participants' judgement of the trustworthiness the people responsible for producing the number was not affected by the inclusion of uncertainty in any form (figure 2*c*).

### 3.3. Interim discussion

Consistent with the UK findings of Study 1, participants in Study 2 perceived information including an explicit verbal uncertainty cue as more uncertain than the same communication without it, and were less trusting of the numbers provided, but did not consider the source of such information less trustworthy. The presence of numeric uncertainty had no significant impact on the perceived trustworthiness of the number or the source. These effects were consistent across messages communicating either current or future uncertainty about COVID-19 deaths.

Interestingly, future predictions were perceived as more uncertain than current estimates for participants reading the control and numeric uncertainty information, although this framing had no impact on participants’ perceived trustworthiness of the numbers or source. We acknowledge that the future and present stimuli differed in terms of the magnitudes communicated. We did not want to mislead participants with inaccurate information in the midst of the pandemic. However, even for those in the control conditions, not being given any cue as to the magnitude of the uncertainty, the uncertainty of the projected future number of deaths was interpreted to be greater than the uncertainty of present number. As the numeric uncertainty range was substantially larger for the figures in the future condition, this confounds any effects of uncertainty type in these groups. However, even though the uncertainty was rated as higher between the future and the present uncertainty groups, this did not appear to translate to lower perceptions of trustworthiness.

## 4. Discussion

Across two studies, using a very salient topic for participants, we broadly find that communications which express statistical uncertainty as a range around a point estimate are not perceived as less trustworthy, nor are the communicators of such uncertainty considered less trustworthy, than those using a point estimate alone.

Only when drawing on the statistical power of a combined sample of more than 10 000 participants (Study 1) do we find a small negative effect of communicating such uncertainty on perceived trustworthiness of the numbers presented. However, even then the perceived trustworthiness of the source of those numbers was not affected. In this regard, our results are consistent with the prior work of van der Bles *et al*. [6] and others [5]. We extend these findings to the context of the COVID-19 pandemic, as well as using a larger and more diverse participant population.

Extending the work to consider uncertainty around future as well as present estimates, we found that although future estimates were (not surprisingly) perceived as more uncertain than present ones in the control and numeric uncertainty conditions, perceived trustworthiness of the estimates was not lower, and was not significantly affected by the addition of a numerical range. The addition of an explicit verbal statement of uncertainty did increase perceived uncertainty in the number and decrease the perceived trustworthiness of it. However, we saw no effects on the perceived trustworthiness of the source of the information. Previous work has also demonstrated no negative effect of communicating future uncertainty on trust [16,52], although research in this area is not consistent, with some studies finding both positive and negative effects on trust [19], and others showing that the nature of trust effects breaks down according to participant characteristics such as education level [53].

First and foremost, we echo previous recommendations that communicators should feel confident that they can use numerical ranges around their point estimates when communicating COVID-19 or other statistics or projections without significantly undermining audience perceptions of trustworthiness [6,54].

We must note that there was variation between countries in Study 1. This was particularly true for how participants rated messages overall; the range in random intercepts suggested the baseline perceived uncertainty and trustworthiness of the numbers and their source differed among countries. Considering the effects of the experimental manipulations on the perceived trustworthiness of the number, however, there was less variation, with random slopes indicating that few countries deviated significantly estimated the fixed effects.

Where there was variation between countries, the reasons for such differences remain unclear. Study 1 was not intended to be a cross-cultural comparison and the number of countries in the dataset does not allow robust quantitative examination of country-level factors [55]. Further research is required to fully understand the inter-country variation observed. Potential reasons for differences between countries include the slightly different meanings or interpretations of translated terms such as ‘uncertainty’ in items [56], the differing impact of, and response to the COVID-19 pandemic across countries at the time of data collection [57], and differing national attitudes and norms regarding risk and uncertainty [58]. Considering the numerous possible sources of variation, it is then surprising that there was so little variation in experimental effects (random slopes) across countries.

South Korea was the one country where the effect of the experimental manipulation on number trustworthiness appeared to deviate from the overall modelled fixed effects: the numeric message appeared to have a larger negative effect on trustworthiness compared to other countries. We note that among the countries surveyed, South Korea ranks very highly on Hofstede's index of Uncertainty Avoidance, which captures ‘the degree to which the members of a society feel uncomfortable with uncertainty and ambiguity’ [59,60]. While it is tempting to consider this a potential moderating factor, we would point out that Japan and France also rank highly, and we do not see a similar pattern effects in these countries. Future research with a larger sample of countries would allow examination of such country-level factors [55].

Regarding verbal expressions of uncertainty, we find mixed results across Studies 1 and 2. Explicitly acknowledging that uncertainty exists, without any quantification, consistently lowered the perceived trustworthiness of the information presented, and did so to a greater degree than messages presenting uncertainty quantified as a range. Such findings align with work on ambiguity aversion [61], which position ambiguity as unmeasured uncertainty in comparison to known uncertainty. All things being equal, people prefer known risks to unknown risks. In the current studies this aversion may underly the lower trustworthiness ratings of the verbal uncertainty message which is patently more ambiguous than the numeric uncertainty message (see also [6]). However only in Study 1 did the verbal uncertainty message also have a negative effect on the perceived trustworthiness of the source of the information. In Study 2, there was no significant effect of verbal uncertainty on source trustworthiness. These results mirror the inconsistent findings of van der Bles *et al*. [6] who found that such verbal uncertainty cues negatively affected perceived trustworthiness of a source in some contexts but not others. Considering these mixed findings we would suggest communicators avoid translating quantified uncertainties into general statements such as ‘there is some uncertainty around this figure’. Further research is needed to clarify the conditions under which verbal uncertainty statements do or do not undermine source trustworthiness.

In no case did we find that expressions of uncertainty *increase* perceived trustworthiness of numbers or their source. This is noteworthy as some previous research has found that some kinds of uncertainty increase the trustworthiness of information and communicators, for example in Earthquake and climate projections [39,62,63]. However other studies have found no effect of uncertainty communication on trustworthiness in other domains (e.g. medicine, climate change [33,64]). These mixed findings are not surprising given the range of issues, forms of uncertainty and operationalizations of trustworthiness and credibility used in previous research [5]. We hope the current study contributes to ongoing work in identifying the broader factors that determine when communication of uncertainty does or does not garner greater trust.

We did not examine individual differences in these studies. It is possible that for certain target audiences' uncertainty information would have different effects on trustworthiness. For example, numeracy may moderate the effect of uncertainty information on perceptions of trustworthiness, though evidence in this regard is limited. One study investigating the communication of uncertainty in medical diagnoses found that low numeracy individuals were more trusting of broad verbal statements than those which included numeric information [65]. Consideration of this should be balanced against the benefits of using numeric expressions of uncertainty for low numeracy audiences. Broadly speaking, communicating numeric uncertainty information, compared with verbal expressions of uncertainty, results in better comprehension and decision-making for low numeracy audiences (reviewed in [37]). We encourage future research to further investigate the impact of such individual differences on perceptions of communicated statistics and associated uncertainty.

Another key conclusion we can draw from the current results is that the temporal framing of statistics (current versus future) did not change the pattern of results. Although our experiments were limited to the communication of COVID-19 statistics, this result suggests that differing timeframes in experimental stimuli between studies are not a major contributor to the heterogeneity of results. More research is needed to confirm this, but the current study is an important first step.

While a single message with uncertainty may not increase perceived trustworthiness, it might buffer against future damage if figures are revised. Batteux *et al*. [27] report that the inclusion of a range (versus point estimate) in COVID-19 vaccine efficacy communications had no immediate impact on trust. However, when participants were later presented with conflicting evidence (updating the previous estimate), those who had first received a point estimate with no uncertainty reported lower perceived trustworthiness of the communicator, compared to those who had received a range. This suggests that communicating uncertainty up front can mitigate later scepticism when scientific evidence is updated. This aligns with related research on intelligence forecasts. In hindsight, decision makers ascribe less blame and more credibility to forecasters who express the probability of an event occurring as range rather than a point estimate [66]. Such findings are particularly relevant to situations such as the COVID-19 pandemic, where evidence and recommendations are frequently updated [28,67]. Further research in this vein is warranted, including examination of boundary conditions. For example if the uncertainty around a statistic is communicated as a range but the actual value is later revealed to be outside that range, does the initial acknowledgement of uncertainty still buttress the communicator's credibility?

Our findings elucidate the effects of numeric uncertainty on trustworthiness, in a broad population sample and two temporal framings, and gives us some confidence in our conclusions. However, we must acknowledge some limitations. Firstly, the uncertainty ranges communicated in our messages were all drawn from existing reports. While this provides some ecological validity (in the sense of Kihlstrom [68]), it did mean we were not able to control for the magnitude of the range presented across some conditions. Previous research suggests people may be less trusting of uncertainty presented as a very large numeric range [6,28,69], although a study similar to those reported here found members of the public were not sensitive to changing magnitudes expressed as a numerical range [6]. More research is needed to identify the effect of changing range magnitudes and how this varies across contexts. We also note that the data collection period for Study 1 occurred over three months, and the infection hospitalization rates reported to the public may have changed over this period, or have varied by local context. We cannot rule out the possibility that this may have influenced some participants' perceptions of the figure presented in the stimuli.

Secondly, Study 2 was only conducted in a UK sample. As shown in Study 1, there is between-country variation in how people respond to uncertainty. Therefore, we cannot necessarily generalize our UK findings to other national contexts.

Third, we included the lexical hedge words ‘around’ or ‘about’ ahead of the core statistic presented, across all conditions in Studies 1 and 2 respectively. The word itself could signal uncertainty to participants [70]. Thus, our control condition might assume a baseline uncertainty around the point estimate and our verbal and numeric statements are adding explicit uncertainty. However, previous research has suggested that when incorporated into phrases such words are unlikely to significantly affect participants' perceptions [6,71].

Fourth, the experiments were embedded in larger surveys and followed a series of questions about the participants' experience with and perceptions of COVID-19. We cannot rule out the possibility that questions posed to participants before the experimental manipulation affected the way that they perceived the materials and answered the questions.

Lastly, the types of uncertainty we examined in the current research were narrowly defined. Thus, our findings may not map onto other forms or formats of uncertainty. For example, statistical uncertainty could be expressed visually, rather than as numerals (e.g. [72,73]), and the existence of unquantified uncertainty could be phrased in a multitude of different ways. Uncertainty can also be further attributed to different sources, with potentially differing impacts. For example, claims could be accompanied by statements about ‘indirect’ uncertainty (in the sense of [9]) due to for instance a lack of data, poor quality research, disagreement among experts, or the nature of the scientific method itself [5]. The inclusion of such explanations may moderate the effects of uncertainty on perceived trustworthiness [40,74].

In conclusion, the studies reported here find that acknowledging uncertainty in the form of a numeric range has minimal impact on the perceived trustworthiness of numbers or their source in the context of COVID-19 statistics, regardless of whether the figures relate to current estimates or future projections. Verbal statements explicitly pointing out the existence of uncertainty around a number, but not quantifying it, decrease trust in the number. Such statements may also undermine the perceived trustworthiness of the source of the information, but we cannot draw strong conclusions in this regard. On that basis we would encourage communicators of uncertainty to, where possible, use numbers, rather than words when communicating uncertainty.

## Data Availability

Data and R analysis scripts are available on the Open Science Foundation website: https://osf.io/y982k/?view_only=07f2c4fd8000488f90f564f5a209bb9c. Supplementary material is available online [75].

## Footnotes

1 Although terms such as 'accurate’, ‘reliable’ and ‘trustworthy’ are treated as conceptually distinct by some scholars, the high inter-item correlations, evidenced by Cronbach's alpha, indicate that these items are essentially measuring the same construct. On this basis, and the use of similar scales in previous research, we treat these items combined as a measure of trustworthiness.
